# Does Sedentary Behavior Context Matter for Problematic Smartphone Use? Associations with Screen-Based, Non-Screen-Based, and Mixed Sedentary Behavior in Adults

**DOI:** 10.3390/bs16101929

**Published:** 2026-10-17

**Authors:** Abdullah Bandar Alansare

**Affiliations:** Department of Exercise Physiology, College of Sports Sciences and Physical Activity, King Saud University, Riyadh 11461, Saudi Arabia; aalansare@ksu.edu.sa; Tel.: +966-555061381

**Keywords:** smartphone use, social media use, mobile phone, technology, sitting

## Abstract

**Background**: This study assessed the associations of screen-based, non-screen-based, and mixed sedentary behavior (SB) with problematic smartphone use (PSU) in adults, and explored whether statistically reallocating screen-based SB to non-screen-based or mixed SB would be associated with lower PSU. **Methods**: This secondary analysis of a web-based cross-sectional study included 1037 adults (average age = 28.9 years; 52.9% women). The culturally adapted Arabic version of the Sedentary Behavior Questionnaire estimated SB. The Arabic version of the Smartphone Addiction Scale measured PSU. Adjusted partition regression and isotemporal substitution models evaluated the study’s objectives. **Results**: Higher screen-based SB was associated with higher PSU (B ranged between 1.725 and 5.891 points; *p* < 0.05 for all). Non-screen-based and mixed SB were associated with lower PSU in specific analyses among women (B ranged between −1.200 and −1.886 points; *p* < 0.05 for all), whereas no statistically significant associations were observed among men (*p* > 0.05). Statistically reallocating time spent in screen-based SB to non-screen-based or mixed SB was associated with lower PSU in several analyses (B ranged between −2.299 and −7.777 points; *p* < 0.05 for all). For weekdays and weekends, statistically significant associations were observed for selected SB categories, with fewer statistically significant associations observed in the weekend analyses. **Conclusions**: These findings suggest that higher screen-based SB was associated with higher PSU, whereas associations of non-screen-based and mixed SB with PSU were less consistent. Statistically reallocating reported screen-based SB to non-screen-based or mixed SB may be associated with lower PSU. These findings do not establish causal effects or actual behavioral changes, and further prospective and experimental research is required.

## 1. Introduction

Smartphones have become integral to modern life, supporting communication and a wide range of personal, health, and economic activities (Ariano et al., 2023; Gómez-Cambronero et al., 2024; Ma et al., 2020; Sun et al., 2023; Yus, 2021). Approximately 5.5 billion people worldwide are estimated to use smartphones in 2026, and this number is expected to continue increasing (Turner, 2023). Although smartphones provide numerous benefits, excessive smartphone use may lead to problematic smartphone use (PSU), which generally refers to problematic patterns of smartphone use characterized by impaired control over smartphone use despite negative consequences (Nawaz, 2024). PSU has attracted increasing public health attention due to its associations with adverse health, well-being, and daily functioning, including impaired sleep quality, poor mental health, reduced professional performance, and family conflict (Busch & McCarthy, 2021; Meng et al., 2022). Importantly, PSU is conceptually distinct from simply spending more time using a smartphone. While smartphone use reflects the amount or frequency of use, PSU reflects maladaptive patterns of use characterized by impaired control and continued engagement despite negative impacts (Nawaz, 2024). Thus, identifying modifiable behavioral contexts associated with impaired control over smartphone use, rather than focusing solely on the amount of smartphone use, may better inform preventive strategies. 

Sedentary behavior (SB), defined as any waking behavior that occurs in a seated, lying, or reclining posture with energy expenditure of ≤1.5 metabolic equivalents (Tremblay et al., 2017), is widespread among adults globally (Bauman et al., 2017). SB has been recognized as an important modifiable risk factor for several psychological disorders, including depression and anxiety (Hallgren et al., 2020) and addiction-like behaviors, such as gaming and gambling (Algren et al., 2015; Chan et al., 2022). Although higher SB has been linked to higher smartphone use among adults (Barkley & Lepp, 2016; Grimaldi-Puyana et al., 2020), SB is a heterogeneous behavior that may differ substantially in its psychological and environmental contexts. For example, sitting while engaging with digital media differs from sitting while reading, doing artwork or crafts, or playing a musical instrument. Consequently, the association of SB with PSU may depend not only on total SB time but also on the type of SB. Evidence directly examining this relationship appears limited, with one recent study reporting that mentally passive SB (i.e., involving minimal cognitive engagement), but not mentally active SB (i.e., involving greater cognitive engagement), was positively associated with PSU in adults (Masoud, 2026). However, the later study examined SB according to its mental/cognitive characteristics and did not distinguish SB according to its screen-related characteristics. Thus, whether SB differs in its associations with PSU according to its screen-based, non-screen-based, or mixed nature remains unclear.

A behavioral reinforcement perspective may help explain why various SB contexts, such as screen-based vs. non-screen-based SB, could have distinct associations with PSU. Screen-based SB may provide repeated opportunities for exposure to digital content, social interaction, entertainment, and other digitally mediated cues and rewards (Wang et al., 2025). Importantly, digital devices are not inherently sedentary or maladaptive, as their implications depend on their purpose and context. Digital technologies can also support activity-oriented approaches, such as physical activity (PA) promoting mobile applications and exergaming (Papale et al., 2025). Thus, screen exposure and SB are related but conceptually distinct constructs. These repeated opportunities may provide conditions associated with habitual smartphone checking and repeated engagement with digital content (Meier, 2022; Oulasvirta et al., 2012). However, some screen-based SB activities directly involve smartphone use, such as watching videos on a smartphone or talking on the phone. Therefore, associations of screen-based SB and PSU may partly reflect greater opportunities for smartphone engagement inherent in these behaviors, rather than sedentary context alone. In contrast, non-screen-based SB, such as doing artwork or crafts or playing a musical instrument, typically involves less direct interaction with digital devices and may therefore provide fewer opportunities for smartphone-related cues and engagement. Consistent with the possibility that different types of SB have distinct psychological correlates, previous studies have reported differences in affective and mental-health outcomes between screen-based and non-screen-based SB (Hudgins et al., 2023; Sadarangani et al., 2023). Moreover, mixed SB may involve sedentary activities incorporating both screen-based and non-screen-based components within the same activity or sedentary period. For instance, some sedentary activities, such as reading and listening, may involve either screen-based or non-screen-based media. As such, examining mixed SB as a distinct category allows this combined SB context to be captured rather than arbitrarily attributing its associations to either screen-based or non-screen-based SB. Thus, whether screen-based, non-screen-based, and mixed SB differ in their associations with PSU remains an important question that has yet to be established. 

Therefore, this secondary analysis addressed a distinct and complementary question to the previous analysis of this dataset (Masoud, 2026). Although the two analyses share the same underlying dataset and PSU outcome, they differ in their exposure classification, conceptual framework, and primary research question. Whereas the previous analysis focused on the mental/cognitive characteristics of SB, the present study focuses on their screen-related characteristics and potential opportunities for smartphone-related engagement. Accordingly, the present study addressed three research questions: (1) Are screen-based, non-screen-based, and mixed SB associated with PSU in adults? (2) Is statistically reallocating reported time from screen-based to non-screen-based or mixed SB associated with PSU while holding total SB constant? (3) Do the observed associations vary descriptively across SB patterns (weekdays vs. weekends) and by sex? It was hypothesized that screen-based SB would be positively associated with PSU, whereas non-screen-based and mixed SB would show weaker or inverse associations with PSU. It was also hypothesized that statistically reallocating reported screen-based SB to non-screen-based or mixed SB would be associated with lower PSU. The weekday/weekend and sex-stratified analyses were interpreted descriptively rather than as formal tests of differences between periods or sexes.

## 2. Materials and Methods

### 2.1. Study Design and Ethics

This secondary analysis used data from a recently published web-based cross-sectional study (Masoud, 2026). A detailed description of the study protocol has been published elsewhere (Masoud, 2026). The Strengthening the Reporting of Observational Studies in Epidemiology (STROBE) Guidelines were used to report this study. 

### 2.2. Participants and Recruitment

The original study used a web-based recruitment strategy to recruit adults from across Saudi Arabia. The survey link was distributed via official King Saud University email lists and widely utilized social media platforms (i.e., Instagram, X, WhatsApp, and Snapchat) (Al Sari et al., 2022). Individuals were eligible if they were adults aged 18 to 65 years, residing in Saudi Arabia, and able to read the Arabic versions of the questionnaires. Initially, 1054 respondents completed all study questionnaires. Of them, 17 individuals were excluded due to missing data or implausible values, resulting in a final analytical sample of 1037 adults.

### 2.3. Measurements

#### 2.3.1. Participants Characteristics

Demographic and general characteristics were self-reported by each participant, including age (years), sex, current marital status, education, current occupation status, last measured height (cm) and weight (kg), and physician-diagnosed chronic diseases. Body mass index (BMI) was calculated as weight (kg) divided by height (m^2^). Participants also reported the time spent in screen-based, non-screen-based, and mixed SB across the week, on weekdays, and on weekends. Overall, participants had a mean age of 28.9 years; 52.9% were women, 66.3% were unmarried, 69.2% had at least an undergraduate degree, and most reported no diagnosed chronic disease (89.3%) (Table 1). Median screen-based SB was 4.3 h/day across the week, on weekdays, and on weekends, whereas median non-screen-based SB was 1.3 h/day across the week, on weekdays, and on weekends. Median mixed SB was 3.0 h/day on weekdays, 2.7 h/day across the week, and 2.0 h/day on weekends. Finally, the median SAS score was 101.0, with scores ranging from 33.0 to 198.0.

#### 2.3.2. Problematic Smartphone Use

The Arabic version of the Smartphone Addiction Scale (SAS), a comprehensive smartphone-specific instrument for assessing PSU, was used to assess PSU (El Keshky et al., 2022; Kwon et al., 2013). The SAS consists of 33 items developed to assess 6 dimensions of PSU, including daily-life disturbance, positive anticipation, withdrawal, cyberspace-oriented relationships, overuse, and tolerance. Each item is rated on a six-point Likert scale ranging from 1 to 6. Item scores were summed to obtain a total SAS score, with higher scores indicating greater PSU. The total score was used as the outcome variable in the present study. The Arabic SAS demonstrated excellent internal consistency in the present sample (Cronbach’s alpha = 0.95).

#### 2.3.3. Sedentary Behavior

The culturally adapted Arabic version of the Sedentary Behavior Questionnaire (SBQ) was used to estimate time spent in screen-based, non-screen-based, and mixed SB (Alaqil et al., 2023; Rosenberg et al., 2010). The SBQ estimates time (hours/day) spent in 9 SB types separately for weekdays and weekends. For each SBQ item, participants reported the time spent in that specific sedentary activity separately for weekdays and weekends. The reported durations were used as activity-specific time estimates for calculating the 3 SB categories. Before statistical analyses, the 9 SB types were prespecified and classified into three researcher-derived analytical categories, including screen-based, non-screen-based, and mixed SB. Screen-based SB encompassed watching TV or videos on a smartphone or tablet while seated, playing electronic and video games while seated, and sitting and talking on the phone. Non-screen-based SB consisted of doing artwork or crafts, playing a musical instrument, and sitting and driving or riding a car, bus, or train. Mixed SB included doing paperwork or computer work (e.g., sending emails or paying bills), sitting and listening to an audio recording on a radio, smartphone, or tablet, and sitting and reading a printed book or magazine or reading from a smartphone or tablet. Mixed SB comprised SB items that may involve either screen-based or non-screen-based components. However, the SBQ does not determine the specific medium used for these SB items. As such, the mixed SB category was retained as a distinct researcher-derived analytical category rather than being interpreted as an empirically measured context involving simultaneous screen-based and non-screen-based SB. Weekday and weekend time for each SB category was calculated by summing the durations of the corresponding SB types. Importantly, each of the nine SBQ items was assigned to only one of the three prespecified categories, such that the categories were mutually exclusive at the level of SBQ activity classification and all nine items were represented. However, the SBQ did not assess whether multiple SB activities were performed concurrently. Hence, the reported durations reflect the time reported for each specific SBQ activity, but the specific medium used for SB items classified as mixed and the extent to which multiple SB activities were performed concurrently cannot be determined from the SBQ. Average daily time across the week was subsequently estimated for each SB category using the following formula: [(weekday SB × 5) + (weekend SB × 2)]/7 (Rosenberg et al., 2010).

#### 2.3.4. Statistical Analysis

Participants’ demographic and general characteristics and SB levels were summarized using descriptive statistics. Continuous variables were summarized using means ± standard deviations (SDs) or medians and interquartile ranges (IQRs), as appropriate, whereas categorical variables were summarized using frequencies and percentages. Partition regression models examined the associations of screen-based, non-screen-based, and mixed SB with PSU in adults, while adjusting for age, BMI, sex, current marital status, education, current occupation status, physician-diagnosed chronic diseases, and the other SB categories. Partition and isotemporal substitution models represent different parameterizations of the same underlying time-use data and address different analytical questions. The partition model estimates the associations of each SB category with PSU while holding the other SB categories constant, whereas the isotemporal substitution parameterizations estimate the associations corresponding to reallocating a fixed amount of time from one SB category to another while holding total SB constant. Total SB was calculated as the sum of screen-based, non-screen-based, and mixed SB. For the statistical reallocation of screen-based SB, the models included total SB, non-screen-based SB, and mixed SB, while screen-based SB was omitted. The model was specified as follows: PSU = B_0_ + B_1_ (total SB) + B_2_ (non-screen-based SB) + B_3_ (mixed SB) + ∑ B_K_ (covariates) + ϵ. Thus, B_2_ represents the estimated difference in PSU associated with statistically reallocating an equivalent amount of time from screen-based SB to non-screen-based SB, whereas B_3_ represents the corresponding reallocation from screen-based SB to mixed SB, while holding total SB, the remaining included SB category, and the specified covariates constant. These coefficients represent statistical reallocations of the reported SB durations and should not be interpreted as observed behavioral changes or intervention effects. In sex stratified analyses, sex was not included as a covariate. Analyses were conducted separately for average daily SB across the week, on weekdays, and on weekends, and repeated for the overall sample and stratified by sex. The significance level was set at *p* < 0.05. All statistical analyses were performed using JASP software (version 0.98.1, JASP Team, University of Amsterdam, The Netherlands).

## 3. Results

Table 2 summarizes the associations of screen-based, non-screen-based, and mixed SB with PSU in adults. Higher screen-based SB accumulated across the week, on weekdays, and on weekends was significantly associated with higher PSU among the overall sample (B ranged from 1.950 to 4.883 points; *p* < 0.05 for all). Mixed SB accumulated on weekdays was inversely associated with PSU (B = −1.200 points; *p* = 0.008). Non-screen-based SB was not associated with PSU (*p* > 0.05 for all).

Among men, higher screen-based SB accumulated across the week and on weekends was associated with higher PSU (B = 3.813 and 2.165 points, respectively; *p* < 0.05 for both). Among women, higher screen-based SB accumulated across the week, on weekdays, and on weekends was associated with higher PSU (B ranged from 1.725 to 5.891 points; *p* < 0.05 for all). Among women, higher non-screen-based SB accumulated across the week and mixed SB on weekdays were inversely associated with PSU (B = −1.886 and −1.228 points, respectively; *p* < 0.05). No other statistically significant associations were observed in the sex-stratified analyses (*p* > 0.05 for all).

Table 3 presents the isotemporal associations of screen-based, non-screen-based, and mixed SB with PSU. Statistically reallocating one hour of screen-based SB to non-screen-based SB across the week, on weekdays, and on weekends was associated with lower SAS scores in the overall sample (B ranged from −2.299 to −5.484 points; *p* < 0.05 for all). Furthermore, statistically reallocating one hour of screen-based SB to mixed SB across the week and on weekdays was associated with lower SAS scores (B = −5.398 and −3.576 points, respectively; *p* < 0.05 for both). Statistically reallocating mixed SB to non-screen-based SB, or vice versa, was not associated with PSU (*p* > 0.05).

Among men, statistically reallocating one hour of screen-based SB to non-screen-based SB across the week was associated with lower PSU (B = −2.827 points; *p* = 0.019). Similarly, statistically reallocating one hour of screen-based SB to mixed SB across the week and on weekdays was associated with lower PSU (B = −4.548 and −2.685 points, respectively; *p* < 0.05 for both). Among women, statistically reallocating one hour of screen-based SB to non-screen-based SB across the week and on weekdays was associated with lower PSU (B = −7.777 and −4.616 points, respectively; *p* < 0.05 for both). Likewise, statistically reallocating one hour of screen-based SB to mixed SB across the week and on weekdays was associated with lower PSU (B = −6.520 and −4.605 points, respectively; *p* < 0.05 for both). No significant associations were observed when statistically reallocating mixed SB to non-screen-based SB (*p* > 0.05).

## 4. Discussion

To the best of our knowledge, this is the first study that simultaneously examined the associations of screen-based, non-screen-based, and mixed SB across the day, on weekdays, and on weekends with PSU in overall adults, men, and women, separately, and explored the associations of statistically reallocating time among these SB types with PSU using isotemporal substitution models. Overall, the findings showed relatively consistent positive associations of screen-based SB with PSU across SB time periods, whereas the inverse associations observed for non-screen-based and mixed SB were less consistent and occurred only in specific analyses. Similarly, statistically reallocating reported screen-based SB to non-screen-based or mixed SB was associated with lower PSU. Statistically significant associations were observed for selected SB categories in the weekday and weekend analyses, with additional associations detected in the weekday analyses. Taken together, these findings suggest that examining different types of SB may provide additional information about their associations with PSU in adults beyond total SB alone, and that statistically reallocating reported screen-based SB to non-screen-based or mixed SB may be associated with lower PSU. Nonetheless, because of the cross-sectional design, the findings should be interpreted with caution, as causal relationships cannot be established. Alternatively, individuals with higher PSU symptom severity may spend more time engaging in smartphone-related SB, such as watching videos or talking on the phone while sitting. As a result, the observed association may reflect, at least in part, reverse causation rather than an effect of screen-based SB on PSU.

Although most adults use their smartphones while sitting (Barkley & Lepp, 2016; Fennell et al., 2019), research examining the association of SB with PSU in adults remains limited (Nambirajan et al., 2025; Sabino et al., 2026). Previous investigations found that higher overall SB was associated with higher smartphone use in adults (Barkley & Lepp, 2016; Grimaldi-Puyana et al., 2020). To the best of our knowledge, only one previous study has specifically assessed the association of SB with PSU in adults, reporting that higher mentally passive SB (i.e., minimally cognitively engaging SB) was associated with higher PSU, whereas mentally active SB (i.e., highly cognitively engaging SB) was not (Masoud, 2026). Extending this literature, the present study suggests that distinguishing between screen-based, non-screen-based, and mixed SB may provide a more nuanced understanding of the relationship between SB and PSU than considering total SB alone, given the contrasting associations observed across these types. Taken together, the available evidence suggests that SB may be an important behavioral factor associated with PSU among adults, and that the type of SB may be more relevant than the overall amount of SB for PSU. However, as the available evidence is based on cross-sectional studies, prospective longitudinal and experimental research is warranted to clarify the temporal and causal relationships between overall and different SB types and PSU in adults.

Two recent systematic reviews reported that higher PA may be associated with lower PSU in adults (Pirwani & Szabo, 2024, 2025). However, one in three adults worldwide is physically inactive (Strain et al., 2024). Thus, alternative behavioral approaches are important for reducing PSU among adults. Herein, the present study suggests that although increasing PA remains an important behavioral target for reducing PSU (Pirwani & Szabo, 2024, 2025), modifying the type of SB may represent an additional behavioral target, particularly in settings where PA is impractical, such as while commuting or waiting. Specifically, the present study indicates that statistically reallocating one hour of screen-based SB to non-screen-based or mixed SB may be associated with lower PSU.

Interestingly, the sex-stratified and weekday/weekend analyses yielded statistically significant associations in selected analyses. Among women, several associations were observed between SB categories and PSU, whereas among men, statistically significant associations were observed for selected screen-based SB measures. These findings should be interpreted descriptively because formal comparisons of regression coefficients between men and women were not performed. One possible explanation for the associations observed among women is that smartphone use patterns and motivations may differ by sex. For instance, women have been reported to use smartphones more frequently for social networking and interpersonal communication (Lenhart et al., 2010; Su et al., 2020) and to report higher levels of PSU than men (Choi et al., 2015). Such differences in smartphone use may potentially contribute to the observed associations, although this possibility was not directly examined in the present study. Likewise, statistically significant associations were observed for selected SB categories in the weekday and weekend analyses, with additional associations detected in the weekday analyses. One possible explanation for the patterns observed on weekdays is that weekdays are generally characterized by more structured daily routines, greater occupational screen exposure, and more consistent SB patterns on weekdays than on weekends (Fukushima et al., 2021; Saidj et al., 2015). Still, these explanations remain speculative and should be examined in future research. Collectively, the available evidence suggests that both increasing PA and reducing SB may be associated with lower PSU among adults. When increasing PA is not feasible, reallocating time from screen-based SB to non-screen-based or mixed SB may be associated with lower PSU.

Several plausible mechanisms may explain the observed associations of different SB types with PSU. Importantly, screen exposure and SB are conceptually distinct, as digital-device use is not inherently sedentary or maladaptive and may serve different purposes and contexts, including activity-oriented applications and exergaming (Papale et al., 2025). Screen-based SB may provide greater opportunities for prolonged exposure to digital screens and repeated smartphone-related cues, such as smartphone notifications, social media updates, and other digital stimuli, all of which may reinforce habitual smartphone use and checking (Nawaz, 2024). Although screen-based SB includes activities beyond smartphone use (e.g., TV viewing and computer use) (Alaqil et al., 2023; Rosenberg et al., 2010), some of these activities may occur within digital environments that provide opportunities for exposure to digital cues and facilitate habitual smartphone engagement. Neuroimaging and behavioral evidence suggest that PSU is characterized by heightened cue reactivity, greater sensitivity to immediate rewards, and reduced executive control, all of which may increase susceptibility to PSU in a screen-rich environment (Wolf et al., 2025). In addition, screen-based SB may involve more opportunities for social networking, leading to increased social gratification, an important motivational factor underlying PSU (Nawaz, 2024). Conversely, non-screen-based SB may generally involve fewer direct opportunities for digital interaction and smartphone-related cues. However, mixed SB may incorporate both screen-based and non-screen-based components within the same sedentary period, and therefore cannot be readily characterized in terms of either higher or lower digital exposure. Instead, the inverse associations observed for mixed SB may reflect broader behavioral context and purpose of these activities, though the present study did not assess the specific mechanisms underlying these associations. As such, these interpretations should be considered tentative and hypothesis-generating.

### 4.1. Strengths and Limitations

This study has several strengths that warrant mentioning. First, the relatively large sample size (n = 1037) improved the precision of the estimated associations (Meyvis & Van Osselaer, 2018). Second, the web-based recruitment strategy was well suited to the study topic (i.e., PSU) and enabled recruitment of adults from different regions of Saudi Arabia, thereby providing geographic diversity within the sample. Third, the assessments captured a broad range of SB types (Alaqil et al., 2023; Rosenberg et al., 2010), allowing separate evaluation of screen-based, non-screen-based, and mixed SB. Finally, PSU was assessed using the SAS (El Keshky et al., 2022; Kwon et al., 2013), a comprehensive, smartphone-specific instrument rather than an adaptation of a general internet addiction measure.

Still, several limitations should be acknowledged. First, the cross-sectional design of the study precludes inferences about causal relationships (Spector, 2019). Second, the convenience-based recruitment strategy and characteristics of the sample may limit the generalizability of the findings to the broader Saudi adult population. The sample was relatively young, more than half of the participants were students, and the sample had a relatively high level of educational attainment. These characteristics may not reflect the demographic and socioeconomic diversity in the Saudi adult population and may have influenced patterns of SB and PSU. As such, the findings should be interpreted primarily in the context of the sample population and should not be assumed to be nationally representative. Moreover, both SB and PSU were assessed using self-reported instruments, making the findings susceptible to recall and reporting bias (Spector, 2019) and potentially resulting in measurement error in estimates of time spent across different SB types (Prince et al., 2020). An additional limitation concerns the researcher-derived classification of the SBQ items into screen-based, non-screen-based, and mixed SB. The mixed SB category included SB items that may involve either screen-based or non-screen-based components. However, the SBQ did not determine the specific medium used for these sedentary activities. Thus, the mixed SB category should not be interpreted as evidence of simultaneous screen-based and non-screen-based activity, and potential exposure misclassification cannot be excluded. In addition, the SBQ assessed the reported duration of individual SB items but did not determine whether multiple SB activities were performed concurrently. Therefore, the reported durations may not necessarily represent mutually exclusive periods of clock time, and the extent of any overlap between different SB activities cannot be determined from the SBQ. This limitation should be considered when interpreting the isotemporal substitution estimates, which represent statistical reallocations of reported SB duration rather than necessarily reflecting actual reallocations of clock time. An additional limitation related to these instruments is the potential behavioral overlap between screen-based SB and PSU. Some screen-based SB assessed in the study, such as watching videos on a smartphone or tablet and talking on the phone while sitting, directly involve smartphone use. Thus, the observed associations of screen-based SB and PSU may partly reflect greater opportunities for smartphone engagement inherent in these behaviors, rather than sedentary contexts alone. Similarly, some mixed SBs involve smartphone or tablet use, which should be considered when interpreting their associations with PSU.

Furthermore, because most participants in the current study did not report physician-diagnosed chronic disease, the generalizability of the findings to populations with a higher prevalence of chronic health conditions may be limited. Residual confounding cannot be excluded because some potentially relevant factors, including psychological distress, sleep, PA, and other socioeconomic characteristics, were not measured and therefore could not be adjusted for in the analyses. Finally, the number of related statistical comparisons may increase the risk of type I error. Although the analyses were prespecified and hypothesis-driven, no formal adjustment for multiple comparisons was applied. Hence, secondary and stratified findings should be interpreted cautiously, with emphasis on the magnitude, precision, and consistency of associations rather than statistical significance alone. Therefore, future prospective and experimental studies using objective measures of SB (e.g., accelerometers), and methods capable of better identifying specific media used and concurrent SB activities, in addition to self-report instruments, and including larger, more demographically diverse, and population-representative samples are warranted to confirm these findings.

### 4.2. Clinical Implications

As digital technologies have become increasingly integrated into daily life, excessive SB and PSU have become increasingly prevalent worldwide (Bauman et al., 2017; Meng et al., 2022). The present study suggests that screen-based SB may be particularly relevant to PSU among adults. Although the findings should not be interpreted as nationally representative or as evidence of a causal relationship, they highlight the potential relevance of screen-related SB in understanding PSU. Furthermore, although these findings reflect statistical rather than observed behavioral substitutions, they suggest that statistically reallocating reported screen-based SB to non-screen-based or mixed SB was associated with lower PSU symptom severity. However, the practical or clinical significance of these differences cannot be established because no minimal clinically important difference for SAS scores has been established. Therefore, these findings should be interpreted cautiously and confirmed in longitudinal and experimental studies before informing behavioral interventions or public health recommendations.

## 5. Conclusions

This study assessed the associations of screen-based, non-screen-based, and mixed SB across the week, on weekdays, and on weekends with PSU in overall adults, men, and women, separately, and explored whether statistically reallocating reported screen-based SB to non-screen-based or mixed SB was associated with lower PSU. Higher screen-based SB was associated with higher PSU, whereas the inverse associations of non-screen-based and mixed SB with PSU were less consistent and were observed in specific analyses, including among women. Statistically reallocating reported screen-based SB to non-screen-based or mixed SB was associated with lower PSU. Statistically significant associations were observed for selected SB categories in both weekday and weekend analyses, with additional associations detected in the weekday analyses. Although these findings do not establish causal effects or actual behavioral changes, they suggest that examining different types of SB may provide additional information about their associations with PSU in adults beyond total SB alone, and that statistically reallocating screen-based SB with non-screen-based or mixed SB may be associated with lower PSU. Yet, further prospective and experimental research is required to determine the causal relationship between these SB types and PSU.

## Figures and Tables

**Table 1:** Demographic and General Characteristics and Screen-based, Non-screen-based, and Mixed SB and PSU Levels of the Participants (n = 1037).

Characteristic	Mean ± SD, Median [IQR], or n (%)
Age (years)	28.9 ± 11.5
Height (cm)	165.6 ± 9.2
Weight (kg)	70.6 ± 18.5
BMI (kg/m^2^)	25.6 ± 5.9
Underweight	89 (8.6)
Normal Weight	423 (40.8)
Overweight	313 (30.2)
Obese	212 (20.4)
Sex	
Men	488 (47.1)
Women	549 (52.9)
Current Marital Status	
Unmarried	688 (66.3)
Married	349 (33.7)
Education	
Diploma Degree or Less	319 (30.8)
Undergraduate Degree	511 (49.3)
Graduate Degree	207 (19.9)
Current Occupational Status	
Student	546 (52.7)
Employed	406 (39.1)
Currently Unemployed	62 (6.0)
Retired	23 (2.2)
Diagnosed with Chronic Disease	
No	926 (89.3)
Yes	111 (10.7)
Screen-based SB (hours/day)	
Across the Week	4.3 [3.7]
On Weekdays	4.3 [4.0]
On Weekends	4.3 [4.3]
Non-screen-based SB (hours/day)	
Across the Week	1.3 [1.6]
On Weekdays	1.0 [1.5]
On Weekends	1.0 [1.5]
Mixed SB (hours/day)	
Across the Week	2.7 [3.6]
On Weekdays	3.0 [4.3]
On Weekends	2.0 [3.0]
SAS score	101.0 [45.0]

BMI; body mass index, cm; centimeter, IQR; interquartile range, n; number of participants, kg/m^2^; kilogram per meter squared, SAS; smartphone addiction scale, SB; sedentary behavior, SD; standard deviation.

**Table 2:** Associations of Screen-based, Non-screen-based, and Mixed SB with PSU in Adults (n = 1037).

Variable	SB Across the Week (hours/day)			SB on Weekdays (hours/day)			SB on Weekends (hours/day)		
	Screen-Based	Non-Screen-Based	Mixed	Screen-Based	Non-Screen-Based	Mixed	Screen-Based	Non-Screen-Based	Mixed
	B ± SE (*p*-Value)								
PSU	Overall Sample (n = 1037) #								
	**4.883 ± 0.401** **(<0.001)**	−0.602 ± 0.692 (0.385)	−0.516 ± 0.383 (0.178)	**2.377 ± 0.491** **(<0.001)**	−0.259 ± 0.970 (0.789)	**−1.200 ± 0.455** **(0.008)**	**1.950 ± 0.470** **(<0.001)**	−0.349 ± 0.911 (0.702)	0.884 ± 0.509 (0.083)
	Men (n = 488) ∞								
	**3.813 ± 0.561** **(<0.001)**	0.986 ± 1.007 (0.328)	−0.735 ± 0.626 (0.241)	1.349 ± 0.701 (0.055)	1.028 ± 1.402 (0.464)	−1.336 ± 0.705 (0.059)	**2.165 ± 0.690** **(0.002)**	0.041 ± 1.250 (0.974)	0.888 ± 0.810 (0.273)
	Women (n = 549) ∞								
	**5.891 ± 0.574** **(<0.001)**	**−1.886 ± 0.953** **(0.048)**	−0.629 ± 0.487 (0.197)	**3.376 ± 0.687** **(<0.001)**	−1.239 ± 1.358 (0.362)	**−1.228 ± 0.595** **(0.039)**	**1.725 ± 0.639** **(0.007)**	−0.733 ± 1.330 (0.582)	0.765 ± 0.663 (0.249)

B; beta coefficient, PSU; problematic smartphone use, SB; sedentary behavior, SE; standard error. Bold indicates significant associations (*p* < 0.05). # indicates models were adjusted for age, BMI, sex, current marital status, education, current occupation status, and physician-diagnosed chronic diseases, and simultaneously adjusted for the other SB. ∞ indicates models were adjusted for age, BMI, current marital status, education, current occupation status, and physician-diagnosed chronic diseases, and simultaneously adjusted for the other SB.

**Table 3:** Isotemporal Associations of Screen-based, Non-screen-based, and Mixed SB with PSU in Adults (n = 1037).

Variable	Overall Sample (n = 1037) #	Men (n = 488) ∞	Women (n = 549) ∞
	B ± SE (*p*-Value)		
Across the Week			
Statistically Reallocating 1 h of Screen-based SB to 1 h of Non-screen-based SB	**−5.484 ± 0.820** **(<0.001)**	**−2.827 ± 1.1201** **(0.019)**	**−7.777 ± 1.119** **(<0.001)**
Statistically Reallocating 1 h of Screen-based SB to 1 h of Mixed SB	**−5.398 ± 0.609** **(<0.001)**	**−4.548 ± 0.918** **(<0.001)**	**−6.520 ± 0.830** **(<0.001)**
Statistically Reallocating 1 h of Mixed SB to 1 h of Non-screen-based SB	−0.086 ± 0.851 (0.920)	1.721 ± 1.306 (0.188)	−1.257 ± 1.132 (0.267)
On Weekdays			
Statistically Reallocating 1 h of Screen-based SB to 1 h of Non-screen-based SB	**−2.636 ± 1.112** **(0.018)**	−0.322 ± 1.633 (0.844)	**−4.616 ± 1.529** **(0.003)**
Statistically Reallocating 1 h of Screen-based SB to 1 h of Mixed SB	**−3.576 ± 0.704** **(<0.001)**	**−2.685 ± 1.052** **(0.011)**	**−4.605 ± 0.953** **(<0.001)**
Statistically Reallocating 1 h of Mixed SB to 1 h of Non-screen-based SB	0.940 ± 1.102 (0.349)	2.364 ± 1.592 (0.138)	−0.011 ± 1.544 (0.994)
On Weekends			
Statistically Reallocating 1 h of Screen-based SB to 1 h of Non-screen-based SB	**−2.299 ± 1.041** **(0.027)**	−2.124 ± 1.456 (0.145)	−2.458 ± 1.501 (0.102)
Statistically Reallocating 1 h of Screen-based SB to 1 h of Mixed SB	−1.066 ± 0.750 (0.155)	−1.277 ± 1.130 (0.259)	−0.960 ± 1.007 (0.341)
Statistically Reallocating 1 h of Mixed SB to 1 h of Non-screen-based SB	−1.233 ± 1.056 (0.243)	−0.847 ± 1.480 (0.567)	−1.497 ± 1.527 (0.327)

B; beta coefficient, SB; sedentary behavior, SE; standard error. Bold indicates significant associations (*p* < 0.05). # indicates models were adjusted for age, BMI, sex, current marital status, education, current occupation status, and physician-diagnosed chronic diseases, and simultaneously adjusted for total SB and the two SB categories included in the model, with the SB category being omitted from the model. ∞ indicates models were adjusted for age, BMI, current marital status, education, current occupation status, and physician-diagnosed chronic diseases, and simultaneously adjusted for total SB and the two SB categories included in the model, with the SB category being omitted from the model.

## Data Availability

The data are available upon request from the corresponding author due to privacy reasons.

## Abbreviations

The following abbreviations are used in this manuscript:

B	Beta Coefficient
BMI	Body Mass Index
IQR	Inter Quartile Range
PSU	Problematic Smartphone Use
SAS	Smartphone Addiction Scale
SB	Sedentary Behavior
SBQ	Sedentary Behavior Questionnaire
SD	Standard Deviation
SE	Standard Error
STROBE	The Strengthening the Reporting of Observational Studies in Epidemiology
